# Listing Occupational Carcinogens

**DOI:** 10.1289/ehp.7047

**Published:** 2004-07-15

**Authors:** Jack Siemiatycki, Lesley Richardson, Kurt Straif, Benoit Latreille, Ramzan Lakhani, Sally Campbell, Marie-Claude Rousseau, Paolo Boffetta

**Affiliations:** ^1^Département de Médecine sociale et préventive, Université de Montréal, Montréal, Québec, Canada; ^2^Department of Epidemiology and Biostatistics, McGill University, Montréal, Québec, Canada; ^3^International Agency for Research on Cancer, Lyon, France; ^4^INRS-Institut Armand-Frappier, Laval, Québec, Canada; ^5^Division of Clinical Epidemiology, German Cancer Research Center, Heidelberg, Germany

**Keywords:** cancer, environment, epidemiology, occupation, review

## Abstract

The occupational environment has been a most fruitful one for investigating the etiology of human cancer. Many recognized human carcinogens are occupational carcinogens. There is a large volume of epidemiologic and experimental data concerning cancer risks in different work environments. It is important to synthesize this information for both scientific and public health purposes. Various organizations and individuals have published lists of occupational carcinogens. However, such lists have been limited by unclear criteria for which recognized carcinogens should be considered occupational carcinogens, and by inconsistent and incomplete information on the occupations and industries in which the carcinogenic substances may be found and on their target sites of cancer. Based largely on the evaluations published by the International Agency for Research on Cancer, and augmented with additional information, the present article represents an attempt to summarize, in tabular form, current knowledge on occupational carcinogens, the occupations and industries in which they are found, and their target organs. We have considered 28 agents as definite occupational carcinogens, 27 agents as probable occupational carcinogens, and 113 agents as possible occupational carcinogens. These tables should be useful for regulatory or preventive purposes and for scientific purposes in research priority setting and in understanding carcinogenesis.

Occupational carcinogens occupy a special place among the different classes of human carcinogens. The occupational environment has been a most fruitful one for investigating the etiology and pathogenesis of human cancer. Up to the 1970s, most recognized human carcinogens were substances or circumstances found primarily in the occupational environment, and although this may no longer be true with the growing list of recognized non-occupational carcinogens, they still represent a large fraction of the total. Although it is important to discover occupational carcinogens for the sake of preventing occupational cancer, the potential benefit of such discoveries goes beyond the factory walls because most occupational exposures find their way into the general environment, sometimes at higher concentrations than in the workplace.

There is a large volume of epidemiologic and experimental data concerning cancer risks in different work environments. It is important to synthesize this information for both scientific and public health purposes. Various national and international bodies have published lists of carcinogens, but available lists of occupational carcinogens have been limited in various ways. Among the issues that are often missing, or treated rather casually, are a coherent assessment of which substances should be considered occupational carcinogens; information on the occupations and industries in which the carcinogenic substances may be found; and the target sites of cancer. The present article represents an attempt to summarize, in tabular form, current knowledge on occupational carcinogens, the occupations and industries in which they are found, and their target organs.

## Methods and Results

### Difficulties in listing occupational carcinogens.

Although it seems like a simple enough task, it is very difficult to draw up an unambiguous list of occupational carcinogens. The first source of ambiguity concerns the definition of an “occupational” carcinogen. Most occupational exposures are also found in the general environment, and/or in consumer products; most general environmental exposures and consumer products, including medications, foods, and others, are found in some occupational environments. The distinctions can be quite arbitrary. For instance, although tobacco smoke, sunlight, and immunosuppressive medications are not primarily considered to be occupational exposures, there certainly are workers whose occupations bring them into contact with these agents. Also, although asbestos, benzene, and radon gas are considered to be occupational carcinogens, they are also found widely among the general population, and indeed, it is likely that many more people are exposed to these substances outside than inside the occupational environment. There is no simple rule to earmark occupational carcinogens as opposed to nonoccupational ones. Further, some carcinogens are chemicals that are used for research purposes and to which few people would ever be exposed, whether occupationally or nonoccupationally. Our operational criterion for designating occupational carcinogens is outlined below.

A second source of ambiguity derives from the rather idiosyncratic nature of the evidence. In some instances, we know that an occupational or industrial group is at excess risk of cancer, and we have a good idea of the causative agent; for example, scrotal cancer among chimney sweeps and polyaromatic hydrocarbons (PAHs) in soot (Waldron 1983), and lung cancer among asbestos miners and asbestos fibers [International Agency for Research on Cancer (IARC) 1977]. In some instances, we know that a group experienced excess risk but the causative agent is unknown or at least unproven [e.g., lung cancer among painters (IARC 1989c), bladder cancer among workers in the aluminum industry (IARC 1987)]. The strength of the evidence for an association can vary. For some associations, the evidence of excess risk seems incontrovertible [e.g., liver angiosarcoma and vinyl chloride monomer (IARC 1979b), bladder cancer and benzidine (IARC 1982b)]. For some associations, the evidence is suggestive [e.g., lung cancer and diesel engine exhaust (IARC 1989a), bladder cancer and employment as a painter (IARC 1989c)]. Among the many substances in the industrial environment for which there are no human data concerning carcinogenicity, there are hundreds that have been shown to be carcinogenic in some animal species and thousands that have been shown to have some effect in assays of mutagenicity or genotoxicity. These considerations complicate the attempt to devise a list of occupational carcinogens.

#### IARC Monographs.

For this task we drew on the authoritative IARC Monograph Program and its evaluation of carcinogenic risks to humans (IARC 1987). The objective of the IARC Monograph Program, which has been operating since 1971, is to publish critical reviews of epidemiologic and experimental data on carcinogenicity for chemicals, groups of chemicals, industrial processes, other complex mixtures, physical agents, and biologic agents to which humans are known to be exposed, to evaluate the data in terms of human risk, and to indicate where additional research efforts are needed.

Substances are selected by IARC for evaluation on the basis of two main criteria: *a*) humans are exposed, and *b*) there is reason to suspect that the substance may be carcinogenic. Direct evidence concerning carcinogenicity of a substance can come from epidemiologic studies among humans or from experimental studies of animals (usually rodents). Additional evidence comes from the results of studies of chemical structure–activity analysis, absorption and metabolism, physiology, mutagenicity, cytotoxicology, and other aspects of toxicity. In the *IARC Monographs*, all types of data contribute to the evaluation.

In this article, we outline the IARC process because it is important to understand how decisions are made in order to properly interpret these decisions. IARC evaluations are carried out during specially convened meetings that typically last a week. The meetings may evaluate only one agent, such as silica, or they may address a set of related agents or even exposure circumstances such as an occupation or an industry. For each such meeting, and there have typically been three per year, IARC convenes an international working group, usually involving from 15 to 30 experts on the topic(s) being evaluated, from four perspectives, *a*) exposure and occurrence of the substances being evaluated, *b*) human evidence of cancer risk (i.e., epidemiology), *c*) animal carcinogenesis, and *d*) other data relevant to the evaluation of carcinogenicity and its mechanisms. The working group is asked to review all of the literature relevant to an assessment of carcinogenicity. In the first part of the meeting, four subgroups (based on the four perspectives mentioned above) review and revise drafts prepared by members of the subgroup, and each subgroup develops a joint review and evaluation of the evidence on which they have focused. Subsequently, the entire working group convenes in plenary and proceeds to derive a joint text. They determine whether the epidemiologic evidence supports the hypothesis that the substance causes cancer, and, separately, whether the animal evidence supports the hypothesis that the substance causes cancer. The judgments are not simply dichotomous (yes/no), but rather they allow the working group to express a range of opinions on each of the dimensions evaluated. Table 1 shows the categories into which the working groups are asked to classify each substance, when examining only the epidemiologic evidence and when examining only the animal experimental evidence. The operational criteria for making these decisions leave room for interpretation, and the scientific evidence itself is open to interpretation. It is not surprising, then, that the evaluations are sometimes difficult and contentious.

For our purpose, there are several limitations to bear in mind. First, IARC does not provide any explicit indication as to whether the substance evaluated should be considered an occupational exposure. Second, although the working groups certainly study the evidence in relation to cancer sites, until recently the formal evaluations did not identify which sites of cancer may be at risk. Site-specific information needs to be gleaned from the working group’s report and other literature. Third, the evaluations are anchored in the time that the working group met and reviewed the evidence; it is possible that evidence appearing after the IARC review could change the evaluation.

### Current knowledge on occupational carcinogens.

From 1972 through 2003, the IARC Monograph Program published 83 volumes, representing evaluations of more than 880 substances, complex mixtures, and industrial processes. Of these, 89 have been classed as definite human carcinogens, 64 as probable, and 264 as possible human carcinogens (IARC 2003). We reviewed each one and earmarked those that we consider to be “occupational exposures.”

In developing a decision rule, we considered the following dimensions: whether the evidence of an effect drew on studies in exposed workers, whether the agent was found more often in the occupational or nonoccupational environments, and the numbers of workers exposed. In the end, the first two dimensions became redundant when we applied the third. Thus, a substance was considered an occupational exposure if there are, or have been, significant numbers of workers exposed to the substance at significant levels. The fact that some workers were exposed to a substance was not enough to label it as an occupational carcinogen. There are many carcinogens to which few workers are exposed, and we did not want to dilute the lists with such obscure agents.

Unfortunately, the knowledge base for determining how many workers are or have been exposed, and at what levels, is very fragmentary. We relied on available documentation such as the *IARC Monographs*, surveys by the National Institute for Occupational Safety and Health (NIOSH 1990), the National Toxicology Program (NTP) *Report on Carcinogens, Tenth Edition* (NTP 2002), and informed guesses on the part of expert industrial hygienists. Where we could come up with approximate numbers of workers exposed, we had to have some type of operational threshold for what should be considered a significant number. As a rule of thumb, we used > 10,000 workers exposed worldwide or > 1,000 in any country, presently or at any time in the past. These were the guidelines against which we measured our imprecise and semisubjective estimates. We also had to operationalize the notion of a level of exposure that was significant. This was even less explicit than the criteria used for numbers of workers exposed; it depended, *inter alia*, on the known range of exposure levels to the agent.

Despite the fact that they may be found in occupational environments, some classes of agents were summarily excluded from consideration on the grounds that the exposures are rare or very infrequent or at very low doses. These included hormones, pharmaceuticals, microbiologic agents, and dietary constituents. Pharmaceuticals represent a special case. Many have been evaluated, and many are considered to be carcinogenic. Although the main population exposed consists of patients undergoing therapy, there can also be exposure of workers who produce the drugs and of health care workers who administer them. But because the exposure doses are orders of magnitude higher among patients than among workers, we have not listed these as occupational carcinogens. Analogously, we have not listed carcinogenic viruses, notably, human immunodeficiency virus (HIV) and hepatitis B and C viruses, although health care workers may be at risk.

With these criteria, we derived the following lists of occupational carcinogens:

- 28 definite human occupational carcinogens (IARC group 1; Table 3)
- 27 probable human occupational carcinogens (IARC group 2A; Table 4)
- 113 possible human occupational carcinogens (IARC group 2B; Table 5)
- 18 occupations and industries that possibly, probably, or definitely entail excess risk of cancer (IARC groups 1, 2A, and 2B; Table 6).

Tables 3–6 only include agents and circumstances that were reviewed and published by the IARC Monograph Program as of 2003. As discussed above, the evaluations are rooted in the information base that was available at the time of the IARC evaluation. As evidence accumulates, the evaluation of an agent can change, as has already occurred in some cases (e.g., cadmium, acrylonitrile). This is why we have included in the tables a reference to the IARC volume in which the substance was evaluated and its date. Evaluations with early dates are more vulnerable to being out of date.

In a special review published in 1987 (Supplement 7), all substances and occupations covered in the first 15 years of the program were reevaluated (IARC 1987). Thus, every substance for which the Supplement 7 reference is cited had an earlier monograph. For many of the substances, there was little, if any, new information, and consequently, we have quoted the original monograph for those without any new data in 1987. For those substances referenced as Supplement 7, new data were available for the reevaluation.

For the agents in Tables 3–5, we devised a set of subheadings to help the reader digest the long lists of often obscure chemical names: physical agents, respirable dusts and fibers, metals and metal compounds, PAHs, wood and fossil fuels and their by-products, monomers, intermediates in plastics and rubber manufacturing, chlorinated hydrocarbons, aromatic amine dyes, azo dyes, intermediates in the production of dyes, pesticides, nitro compounds, and others. Tables 3–5 indicate some of the main occupations or industries in which each listed substance is found, and the strength of evidence from human and animal studies. In Tables 3 and 4, we show the type(s) of cancer affected, with an indication of the strength of evidence for each type listed. Information on target organ is not shown in Table 5 because, for agents listed as possible carcinogens, evidence concerning humans is either conflicting or not available at all.

For many of the agents listed, but not all, there has been some epidemiologic evidence of carcinogenicity among exposed workers. For most of the agents listed, but not all, the occupational environment represents the most common locale of exposure. The most prominent exceptions to this rule are aflatoxins, sunlight, involuntary tobacco smoking, and radon. Whether these cause more cases of cancer as a result of occupational or nonoccupational exposure depends on numbers exposed and exposure levels in the two types of milieu. It is plausible that there may be more cases resulting from nonoccupational exposure.

The IARC Monograph Program has occasionally addressed cancer risk in various occupations and industries, as well as agents. However, although the monograph program aims at a systematic evaluation of agents and complex mixtures, it is not intended to provide a systematic review of cancer risk by industries and occupations. That is, those reviews were conducted where there were particular concerns or anticipated insights regarding specific potential carcinogens. Sometimes this was done when there appeared to be strong evidence of risk in an occupation but little indication of what the responsible agent might be (e.g., rubber industry, painters). Sometimes the impetus for an occupation or industry review came from the attempt to evaluate some agent, but it was realized that the evidence regarding that agent was rooted in epidemiologic evidence regarding some occupation or industry (e.g., glass industry, hairdresser). Table 6 shows those occupations and industries that IARC has evaluated as definitely, probably, or possibly entailing a carcinogenic risk. Because there has been no pretense of exhaustiveness in evaluating occupations and industries, the absence of an occupation or industry in Table 6 does not carry the same significance as the absence of an agent in Tables 3–5. That is, it does not signify that there is no known risk for that occupation or industry.

Because our inclusion criteria admitted substances to which workers were exposed in the past, we included some substances that have been banned or virtually eliminated in some countries, such as mustard gas, bis(chloromethyl) ether, tris(2,3-dibromopropyl) phosphate, and 4,4′-methylene bis(2-chloroaniline) (MOCA), as well as some industries that no longer exist (viz., production of auramine and magenta). These are mentioned partly for historic interest and partly because it is possible that these might yet be used in some places at some time.

It is important to note that the substances, occupations, and industries listed in Tables 3–6 are not mutually exclusive. Certainly, some of the occupations and industries listed in Table 6 may be there because of some of the substances that are listed in Tables 3–5. But further, the substances relate to each other in complicated ways. Some families of substances include some specific substances that are also listed (e.g., nonarsenical insecticides, which includes DDT; benzidine-based dyes, which includes benzidine). Also, there are some complex mixtures (e.g., diesel exhaust) that contain a substance on the list (e.g., nitro-PAHs) that may be responsible for the carcinogenicity of the mixture.

The listing of affected cancer sites in Tables 3 and 4 does not come explicitly from the *IARC Monographs*. Sometimes the affected target organ(s) was rather evident, but sometimes it required that we evaluate the evidence, including evidence published more recently than the IARC evaluation in question. Table 7 shows the same agents listed in Tables 3 and 4 but organized by site of cancer. Again, we indicate clearly which associations are strong and which are only suggestive. The lung is the target organ that has most often been linked to occupational carcinogens.

### The evolution of knowledge.

In order to appreciate how knowledge has evolved, we searched for information on the current occupational carcinogens at two earlier time periods. As mentioned above, IARC carried out a comprehensive cumulative synthesis in 1987 (IARC 1987). In that report, the results were presented with the same rating system (group 1, 2A, 2B, 3) as is used today, rendering the lists comparable. In 1964, even before the establishment of IARC, the World Health Organization (WHO) commissioned an expert panel to survey available knowledge on human carcinogens (WHO 1964). In the WHO report, there was no explicit rating system. It was a discursive presentation of knowledge and opinions that we attempted, with some license, to translate into a simple system corresponding to definite, probable/possible, or not mentioned. From these two reports, we searched for references to the 168 substances presented in Tables 3–5 and that are currently considered to be definite, probable, or possible occupational carcinogens.

Table 8 shows how the current occupational carcinogens were considered in two earlier times. Half of today’s recognized definite occupational carcinogens were already recognized as such by 1964, in the early period of cancer epidemiology. Nearly 90% were considered to be definite or probable as of 15 years ago. In contrast, > 95% of today’s probable and possible occupational carcinogens had not even been mentioned as of 1964, and about one-third were not mentioned as of 1987. Although it is possible for the classification of agents to change over time in either direction, in practice there have been rather few instances of agents being “downgraded” between successive periods. Notable counterexamples include the following:

- 3,3-Dichlorobenzene, which was considered a definite carcinogen in 1964 but was only considered as a possible carcinogen as of 1987 and 2002
- Acrylonitrile and propylene oxide, which were considered probable carcinogens in 1987, but only as possible carcinogens in 2002
- Glass wool was considered a possible carcinogen in 1988 but was downgraded to unclassifiable in 2002
- Ionizing radiation, a special case, was considered a definite carcinogen in 1964 and is so considered today, but it had not been reviewed by IARC before the 1990s; therefore, we had to classify it as “unrated” in 1987.

## Discussion

Many of the recognized definite occupational carcinogens were first suspected before the era of modern epidemiology (i.e., before 1950). The significance of this observation is unclear. It may be that there were only a limited number of strong occupation–cancer associations, and these were sufficiently obvious that they could produce observable clusters of cases for astute clinicians to notice. It may be that levels of exposure to occupational chemicals were so high before the 1950s as to produce high cancer risks and cancer clusters, but that improvements in industrial hygiene in industrialized countries have indeed decreased risks to levels that are difficult to detect. The number of occupational agents rated by IARC as group 1 carcinogens has tapered off since 1987, whereas the proportion of group 2B evaluations has increased. This reflects the fact that, when the monograph program began, there was a “backlog” of agents for which strong evidence of carcinogenicity had accumulated, and, naturally, these were the agents that IARC initially selected for review. Once the agents with strong evidence had been dealt with, IARC started dealing with others. It would be wrong to infer that the historic trend in IARC designations signals that we are approaching the end of the period of potential to discover occupational carcinogens. There are many thousands of chemicals in workplaces, and new ones are continuously being introduced. Most recognized occupational carcinogens were first suspected on the basis of case reports by clinicians or pathologists (Doll 1975). These discoveries were usually coincidental (Siemiatycki et al. 1981). It is thus reasonable to suspect that there may be some, perhaps many, as yet undiscovered occupational carcinogens. Only a small fraction of occupational agents have been adequately investigated with epidemiologic data. There are many reasons for this including, *inter alia*, the magnitude of the numbers of agents to be investigated, a shift away from occupational cancer research in the epidemiologic community and into new areas of epidemiologic interest, the difficulty and challenge of exposure assessment, and increasing barriers to accessing human subjects for occupational studies. These are problems that deserve attention, or we will fail in our responsibilities.

Many countries have agencies that list carcinogens. In the United States the two primary sources of information on occupational carcinogens, at least in the form of lists, are NIOSH and the NTP. NIOSH publishes a list of agents that it considers to be occupational carcinogens (NIOSH 2004). Currently there are 133 agents on this list. There is no further information in the NIOSH list regarding the degree of evidence for different agents, the occupations where these may occur or on the target organs, or the criteria and methods used to establish and update this list. The NTP has been mandated under the Public Health Service Act (1978) to maintain a list of human carcinogens and to provide data on each one concerning exposure circumstances and regulatory policies (NTP 2002). This list uses a two-category scale: “known to be a human carcinogen” and “reasonably anticipated to be a human carcinogen.” Currently, there are 52 agents listed in the first category and 176 in the second. Information concerning each agent is described in a brief report that includes some exposure data as well as health effects data and regulatory data (NTP 2002). The substances on these lists are not limited to occupational agents, and there is no tabular summary of occupational agents, the occupations in which these may occur, or the target organs. It is beyond the scope of this article to carry out a comparison of the procedures and lists of the various national bodies. Suffice it to say that most of them draw heavily on the IARC program and adapt it to their purposes.

There is sometimes a tendency to interpret tables of carcinogens in too categorical a fashion. Although it may be convenient for lobbyists and regulators to divide the world of chemicals and occupational circumstances into “good guys” and “bad guys,” such a dichotomy is simplistic. The determination that a substance or circumstance is carcinogenic depends on the strength of evidence at a given point in time. The evidence is sometimes clear-cut (which would correspond to evaluations of group 1 or group 4), but more often it is not. The balance of evidence can change in either direction as new data emerge.

The characterization of an occupation or industry group as a “high-risk group” is strongly rooted in time and place. For instance, the fact that some groups of nickel refinery workers experienced excess risks of nasal cancer does not imply that all workers in all nickel refineries will be subject to such risks. The particular circumstances of the industrial process, raw materials, impurities, and control measures may produce risk in one nickel refinery but not in another or in one historic era but not in another. The same can be said of rubber production facilities, aluminum refineries, and other industries and occupations. Labeling a chemical substance as a carcinogen in humans is a more timeless statement than labeling an occupation or industry as a high-risk group. However, even such a statement requires qualification. Different carcinogens produce different levels of risk, and for a given carcinogen there may be vast differences in the risks incurred by different people exposed under different circumstances. Indeed, there may be threshold effects or interactions with other factors, environmental or genetic, that produce no risk for some exposed workers and high risk for others.

This raises the issue of quantitative risk assessment, which is an important tool in prevention of occupational cancer. Unfortunately, our tables provide no basis for gauging the strength of the effect of each carcinogen, either in relative risk terms or in absolute risk terms, or in terms of dose–response relationships. The IARC evaluations provide no such indications, and although it would be most desirable to have such information, for most agents the information base to support such quantification is fragmentary.

In summary, the listing of occupational carcinogens is important. It provides a yardstick of our knowledge base, it provides guidance in setting research priorities, and it provides an important tool for prevention of cancer. Regulatory procedures and other aspects of cancer prevention depend on the listing of carcinogens. The IARC Monograph Program has been an indispensable component of this process. The tables presented herein, based on *IARC Monographs* but augmented in various ways, will be useful to researchers in setting research priorities and in furthering our understanding of carcinogenesis, and to those interested in preventing occupational cancer.

## Figures and Tables

**Table 1:** Classifications used in the *IARC Monographs* to characterize evidence of carcinogenicity.

Category of evidence	In humans	In animals
Sufficient evidence of carcinogenicity	A causal relationship has been established between exposure to the agent, mixture, or exposure circumstances and human cancer. That is, a positive relationship has been observed between the exposure and cancer in studies in which chance, bias, and confounding could be ruled out with reasonable confidence.	A causal relationship has been established between the agent or mixture and an increased incidence of malignant neoplasms or of an appropriate combination of benign and malignant neoplasms in *a*) two or more species of animals or *b*) in two or more independent studies in one species carried out at different times or in different laboratories or under different protocols.
Limited evidence of carcinogenicity	A positive association has been observed between exposure to the agent, mixture, or exposure circumstance and cancer for which a causal interpretation is considered to be credible, but chance, bias, or confounding could not be ruled out with reasonable confidence.	The data suggest a carcinogenic effect but are limited for making a definitive evaluation because, for example, *a*) the evidence of carcinogenicity is restricted to a single experiment; *b*) there are unresolved questions regarding the adequacy of the design, conduct, or interpretation of the study; or *c*) the agent or mixture increases the incidence only of benign neoplasms or lesions of uncertain neoplastic potential, or of certain neoplasms that may occur spontaneously in high incidences in certain strains.
Insufficient evidence of carcinogenicity	The available studies are of insufficient quality, consistency, or statistical power to permit a conclusion regarding the presence or absence of a causal association between exposure and cancer, or no data on cancer in humans are available.	The studies cannot be interpreted showing either the presence or absence of a carcinogenic effect because of major qualitative or quantitative limitations, or no data on cancer in experimental animals are available.
Evidence suggesting lack of carcinogenicity	There are several adequate studies covering the full range of levels of exposure that human beings are known to encounter, which are mutually consistent in not showing a positive association between exposure to the agent, mixture, or exposure circumstance and any studied cancer at any observed level of exposure.	Adequate studies involving at least two species are available which show that, within the limits of the tests used, the agent or mixture is not carcinogenic.

**Table 2:** Guidelines used by the IARC Monographs Program in evaluating human carcinogenicity based on the synthesis of epidemiologic, animal, and other evidence.*^a^*

		Combinations that fit in this group		
Group	Description of group	Epidemiologic evidence	Animal evidence	Other evidence
1	The agent, mixture, or exposure circumstance is carcinogenic to humans	Sufficient	Any	Any
		Less than sufficient	Sufficient	Strongly positive
2A	The agent, mixture, or exposure circumstance is probably carcinogenic to humans	Limited	Sufficient	Less than strongly positive
		Inadequate or not available	Sufficient	Strongly positive
2B	The agent, mixture, or exposure circumstance is possibly carcinogenic to humans	Limited	Less than sufficient	Any
		Inadequate or not available	Sufficient	Less than strongly positive
		Inadequate or not available	Limited	Strongly positive
3	The agent, mixture, or exposure circumstance is not classifiable as to its carcinogenicity to humans	Inadequate or not available	Limited	Less than strongly positive
			Not elsewhere classified	
4	The agent, mixture, or exposure circumstance is probably not carcinogenic to humans	Suggesting lack of carcinogenicity	Suggesting lack of carcinogenicity	Any
		Inadequate or not available	Suggesting lack of carcinogenicity	Strongly negative

a This table shows our interpretation of the IARC Monographs Program guidelines to derive the overall evaluation from the combined epidemiologic, animal, and other evidence. However, the IARC working groups can, under exceptional circumstances, depart from these guidelines in deriving the overall evaluation (IARC 2003s). For example, the overall evaluation can be downgraded if there is less than sufficient evidence in humans and strong evidence that the mechanism operating in animals is not relevant to humans.

**Table 3:** Substances and mixtures that have been evaluated by IARC as definite (group 1) human carcinogens and that are occupational exposures.

Substance or mixture	Occupation or industry in which the substance is found*^a^*	*IARC Monograph* volume (year)*^b^*	Human evidence*^c^*	Animal evidence*^c^*	Site(s)
Physical agents					
Ionizing radiation and sources thereof, including, notably, X rays, γ rays, neutrons, and radon gas	Radiologists; technologists; nuclear workers; radium-dial painters; underground miners; plutonium workers; cleanup workers following nuclear accidents; aircraft crew	Vol. 75 (2000a)	Sufficient	Sufficient	Bone*^d^*
		Vol. 78 (2001a)			Leukemia*^d^*
					Lung*^d^*
					Liver*^d^*
					Thyroid*^d^*
					Others*^d^*
Solar radiation	Outdoor workers	Vol. 55 (1992b)	Sufficient	Sufficient	Melanoma*^d^*
					Skin*^d^*
Respirable dusts and fibers					
Asbestos	Mining and milling; by-product manufacture; insulating; shipyard workers; sheet-metal workers; asbestos cement industry	Suppl. 7 (1987)	Sufficient	Sufficient	Lung*^d^*
					Mesothelioma*^d^*
					Larynx*^e^*
					GI tract*^e^*
Erionite	Waste treatment; sewage; agricultural waste; air pollution control systems; cement aggregates; building materials	Suppl. 7 (1987)	Sufficient	Sufficient	Mesothelioma*^d^*
Silica, crystalline	Granite and stone industries; ceramics, glass, and related industries; foundries and metallurgical industries; abrasives; construction; farming	Vol. 68 (1997b)	Sufficient	Sufficient	Lung*^d^*
Talc containing asbestiform fibers	Manufacture of pottery, paper, paint, and cosmetics	Suppl. 7 (1987)	Sufficient	Inadequate	Lung*^d^*
					Mesothelioma*^d^*
Wood dust	Logging and sawmill workers; pulp and paper and paperboard industry; woodworking trades (e.g., furniture industries, cabinetmaking, carpentry and construction); used as filler in plastic and linoleum production	Vol. 62 (1995b)	Sufficient	Inadequate	Nasal cavities and paranasal sinuses*^d^*
Metals and metal compounds					
Arsenic and arsenic compounds	Nonferrous metal smelting; production, packaging, and use of arsenic-containing pesticides; sheep dip manufacture; wool fiber production; mining of ores containing arsenic	Suppl. 7 (1987)	Sufficient	Limited	Skin*^d^*
					Lung*^d^*
					Liver (angiosarcoma)*^e^*
Beryllium	Beryllium extraction and processing; aircraft and aerospace industries; electronics and nuclear industries; jewelers	Vol. 58 (1993a)	Sufficient	Sufficient	Lung*^d^*
Cadmium and cadmium compounds	Cadmium-smelter workers; battery production workers; cadmium-copper alloy workers; dyes and pigments production; electroplating processes	Vol. 58 (1993a)	Sufficient	Sufficient	Lung*^d^*
Chromium compounds, hexavalent	Chromate production plants; dyes and pigments; plating and engraving; chromium ferro-alloy production; stainless-steel welding; in wood preservatives; leather tanning; water treatment; inks; photography; lithography; drilling muds; synthetic perfumes; pyrotechnics; corrosion resistance	Vol. 49 (1990a)	Sufficient	Sufficient	Lung*^d^*
					Nasal sinuses*^e^*
Selected nickel compounds, including combinations of nickel oxides and sulfides in the nickel refining industry	Nickel refining and smelting; welding	Vol. 49 (1990a)	Sufficient	Sufficient	Lung*^d^*
					Nasal cavity and sinuses*^d^*
Wood and fossil fuels and their by-products					
Benzene	Production; solvents in the shoe production industry; chemical, pharmaceutical, and rubber industries; printing industry (rotogravure plants, bindery departments); gasoline additive	Suppl. 7 (1987)	Sufficient	Limited	Leukemia*^d^*
Coal tars and pitches	Production of refined chemicals and coal tar products (patent-fuel); coke production; coal gasification; aluminum production; foundries; road paving and construction (roofers and slaters)	Suppl. 7 (1987)	Sufficient	Sufficient	Skin*^d^*
					Lung*^e^*
					Bladder*^e^*
Mineral oils, untreated and mildly treated	Production; used as lubricant by metal workers, machinists, engineers; printing industry (ink formulation); used in cosmetics, medicinal and pharmaceutical preparations	Suppl. 7 (1987)	Sufficient	Inadequate	Skin*^d^*
					Bladder*^e^*
					Lung*^e^*
					Nasal sinuses*^e^*
Shale oils or shale-derived lubricants	Mining and processing; used as fuels or chemical-plant feedstocks; lubricant in cotton textile industry	Suppl. 7 (1987)	Sufficient	Sufficient	Skin*^d^*
Soots	Chimney sweeps; heating-unit service personnel; brick masons and helpers; building demolition workers; insulators; firefighters; metallurgical workers; work involving burning of organic materials	Vol. 35 (1985)	Sufficient	Inadequate	Skin*^d^*
					Lung*^d^*
					Esophagus*^e^*
Monomers					
Vinyl chloride	Production; production of polyvinyl chloride and co-polymers; refrigerant before 1974; extraction solvent; in aerosol propellants	Suppl. 7 (1987)	Sufficient	Sufficient	Liver (angiosarcoma)*^d^*
					Liver (hepatocellular)*^e^*
Intermediates in plastics and rubber manufacturing					
Bis(chloromethyl) ether and chloromethyl methyl ether (technical grade)	Production; chemical intermediate; alkylating agent; laboratory reagent; plastic manufacturing; ion-exchange resins and polymers	Suppl. 7 (1987)	Sufficient	Sufficient	Lung (oat cell)*^d^*
Aromatic amine dyes					
4-Aminobiphenyl	Production; dyestuffs and pigment manufacture	Suppl. 7 (1987)	Sufficient	Sufficient	Bladder*^d^*
Benzidine	Production; dyestuffs and pigment manufacture	Suppl. 7 (1987)	Sufficient	Sufficient	Bladder*^d^*
2-Naphthylamine	Production; dyestuffs and pigment manufacture	Suppl. 7 (1987)	Sufficient	Sufficient	Bladder*^d^*
Pesticides					
Ethylene oxide	Production; chemical industry; sterilizing agent (hospitals, spice fumigation)	Vol. 60 (1994)	Limited	Sufficient	Leukemia*^d^*
2,3,7,8-Tetrachlorodibenzo-*para*-dioxin (TCDD)	Production; use of chlorophenols and chlorophenoxy herbicides; waste incineration; PCB production; pulp and paper bleaching	Vol. 69 (1997a)	Limited	Sufficient	All sites combined*^d^*
					Lung*^e^*
					Non-Hodgkin lymphoma*^e^*
					Sarcoma*^e^*
Others					
Aflatoxin	Feed production industry; workers loading and unloading cargo; rice and maize processing	Vol. 82 (2002b)	Sufficient	Sufficient	Liver*^d^*
Involuntary (passive) smoking	Workers in bars and restaurants; office workers	Vol. 83 (2004)	Sufficient	Sufficient	Lung*^d^*
Mustard gas	Production; used in research laboratories; military personnel	Suppl. 7 (1987)	Sufficient	Limited	Larynx*^d^*
					Lung*^e^*
					Pharynx*^e^*
Strong inorganic-acid mists containing sulfuric acid	Pickling operations; steel industry; petrochemical industry; phosphate acid fertilizer manufacturing	Vol. 54 (1992a)	Sufficient	Not available	Larynx*^d^*
					Lung*^e^*

a Not necessarily an exhaustive list of occupations/industries in which this agent is found; not all workers in these occupations/industries are exposed. The term “production” is used to indicate that this substance is man-made and that workers may be exposed in the production process.

b Most recent IARC evaluation; for those referenced to Supplement 7 (IARC 1987), it is possible that the 1987 review was quite perfunctory and that the essential evidence was cumulated at an earlier date.

c As judged by the IARC working group; we added the notation “not available” to signify those substances for which there was no evidence at all.

d We judged that evidence for an association with this site was strong.

e We judged that evidence was suggestive.

**Table 4:** Substances and mixtures that have been evaluated by IARC as probable (group 2A) human carcinogens and that are occupational exposures.

Substance or mixture	Occupation or industry in which the substance is found*^a^*	*IARC Monograph* volume (year)*^b^*	Human evidence*^c^*	Animal evidence*^c^*	Site(s)
Physical agents					
Ultraviolet radiation (A, B, and C) from artificial sources	Arc welding; industrial photoprocesses; sterilization and disinfection; phototherapy; operating theaters; research laboratories; ultraviolet fluorescence in food industry; insect traps	Vol. 55 (1992b)	Inadequate	Sufficient	Melanoma*^d^*
Polyaromatic hydrocarbons					
Benz[*a*]anthracene	Work involving combustion of organic matter; foundries; steel mills; firefighters; vehicle mechanics	Vol. 32 (1983b)	Not available	Sufficient	Lung*^d^*
					Bladder*^d^*
					Skin*^d^*
Benzo[*a*]pyrene	Work involving combustion of organic matter; foundries; steel mills; firefighters; vehicle mechanics	Vol. 32 (1983b)	Not available	Sufficient	Lung*^d^*
					Bladder*^d^*
					Skin*^d^*
Dibenz[*a,h*]anthracene	Work involving combustion of organic matter; foundries; steel mills; firefighters; vehicle mechanics	Vol. 32 (1983b)	Not available	Sufficient	Lung*^d^*
					Bladder*^d^*
					Skin*^d^*
Wood and fossil fuels and their by-products					
Creosotes	Brickmaking; wood preserving	Vol. 35 (1985)	Limited	Sufficient	Skin*^d^*
Diesel engine exhaust	Railroad workers; professional drivers; dock workers; mechanics	Vol. 46 (1989a)	Limited	Sufficient	Lung*^d^*
					Bladder*^d^*
Intermediates in plastics and rubber manufacturing					
4,4′-Methylene bis(2-chloroaniline)	Production; curing agent for roofing and wood sealing	Vol. 57 (1993b)	Inadequate	Sufficient	Bladder*^d^*
Styrene-7,8-oxide	Production; styrene glycol production; perfume preparation; reactive diluent in epoxy resin formulations; as chemical intermediate for cosmetics, surface coating, and agricultural and biological chemicals; used for treatment of fibers and textiles; in fabricated rubber products	Vol. 60 (1994)	Inadequate	Sufficient	
Chlorinated hydrocarbons					
α-Chlorinated toluenes	Production; dye and pesticide manufacture	Vol. 71 (1999a)	Limited	Sufficient	Lung*^d^*
Polychlorinated biphenyls	Production; electrical capacitor manufacturing	Suppl. 7 (1987)	Limited	Sufficient	Liver and biliary
					tract*^d^*
Tetrachloroethylene	Production; dry cleaning; metal degreasing	Vol. 63 (1995a)	Limited	Sufficient	Cervix*^d^*
					Esophagus*^d^*
					Non-Hodgkin lymphoma*^d^*
Trichloroethylene	Production; dry cleaning; metal degreasing	Vol. 63 (1995a)	Limited	Sufficient	Liver and biliary tract*^d^*
					Non-Hodgkin lymphoma*^d^*
					Renal cell*^d^*
Monomers					
Acrylamide	Chemical industry; water and wastewater treatment; textile, steel, and lumber industries; petroleum refining; mineral processing; sugar production; hospitals	Vol. 60 (1994)	Inadequate	Sufficient	Pancreas*^d^*
1,3-Butadiene	Chemical and rubber industries	Vol. 71 (1999a)	Limited	Sufficient	Lymphohematopoietic*^d^*
Epichlorohydrin	Production and use of resins, glycerine, and propylene-based rubbers; used as a solvent	Vol. 71 (1999a)	Inadequate	Sufficient	Lung*^d^*
					CNS*^d^*
Vinyl bromide	Production; production of vinyl bromide polymers and monoacrylic fibers for carpet backing material; rubber and plastic production	Vol. 71 (1999a)	Not available	Sufficient	
Vinyl fluoride	Production; polyvinyl fluoride and fluoropolymer production	Vol. 63 (1995a)	Not available	Sufficient	
Aromatic amine dyes					
Benzidine-based dyes	Production; used in textile, paper, leather, rubber, plastics, printing, paint, and lacquer industries	Suppl. 7 (1987)	Inadequate	Sufficient	Bladder*^d^*
4-Chloro-*ortho*-toluidine	Dye and pigment manufacture; textile industry	Vol. 77 (2000b)	Limited	Sufficient	Bladder*^d^*
*ortho*-Toluidine	Production; manufacture of dyestuffs, pigments, optical brightener, pharmaceuticals, and pesticides; rubber vulcanizing; clinical laboratory reagent; cleaners and janitors	Vol. 77 (2000b)	Limited	Sufficient	Bladder*^d^*
Intermediates in the production of dyes					
Dimethylcarbamoyl chloride	Production; manufacture of pharmaceuticals, pesticides, and dyes	Vol. 71 (1999a)	Inadequate	Sufficient	
Pesticides					
Captafol	Production; fungicide	Vol. 53 (1991b)	Not available	Sufficient	
Ethylene dibromide	Production; pest control; petroleum refining and waterproofing; leaded gasoline additive; chemical intermediate and solvent in gums, waxes, resins, dyes, and pharmaceutical preparations	Vol. 71 (1999a)	Inadequate	Sufficient	
Nonarsenical insecticides	Production; pest control and agricultural workers; flour and grain mill workers	Vol. 53 (1991b)	Limited	Not available	Brain*^d^*
					Leukemia*^d^*
					Lung*^d^*
					Multiple myeloma*^d^*
					Non-Hodgkin lymphoma*^d^*
Others					
Diethyl sulfate	Ethanol production	Vol. 71 (1999a)	Not available	Sufficient	
Formaldehyde	Production; pathologists; medical laboratory technicians; plastics; textile industry	Vol. 62 (1995b)	Limited	Sufficient	Leukemia*^d^*
					Nasal sinuses*^d^*
					Nasopharynx*^d^*
Tris(2,3-dibromopropyl)	Production; used in the textile phosphate industry; in phenolic resins (for electronics industry), paints, paper coatings, and rubber	Vol. 71 (1999a)	Inadequate	Sufficient	

CNS, central nervous system.

a Not necessarily an exhaustive list of occupations/industries in which this agent is found; not all workers in these occupations/industries are exposed. The term “production” is used to indicate that this substance is man-made and that workers may be exposed in the production process.

b Most recent IARC evaluation; for those referenced as Supplement 7 (IARC 1987), it is possible that the 1987 review was quite perfunctory and that the essential evidence was cumulated at an earlier date.

c As judged by the IARC working group; we added the notation “not available” to signify those substances for which there was no epidemiologic evidence at all.

d We judged that the evidence was suggestive.

**Table 5:** Substances and mixtures that have been evaluated by IARC as possible (group 2B) human carcinogens and that are occupational exposures.

Substance or mixture	Occupation or industry in which the substance is found*^a^*	*IARC Monograph* volume (year)*^b^*	Human evidence*^c^*	Animal evidence*^c^*
Respirable dusts and fibers				
Glass wool	Production; construction and insulation	Vol. 81 (2002a)	Inadequate	Sufficient
Palygorskite (long fibers > 5 μm)	Miners and millers; production of waste absorbents, fertilizers, and pesticides	Vol. 68 (1997b)	Inadequate	Sufficient
Refractory ceramic fibers	Production; furnace insulators; ship builders; heat-resistant fabric manufacture	Vol. 81 (2002a)	Inadequate	Sufficient
Rock wool	Production; thermal or acoustical insulation	Vol. 81 (2002a)	Inadequate	Limited
Slag wool fireproofing	Production; thermal or acoustical insulation	Vol. 81 (2002a)	Inadequate	Limited
Special-purpose glass fibers such as E-glass and “475” glass fibers	Reinforced plastic industry	Vol. 81 (2002a)	Not available	Sufficient
Metals and metal compounds				
Antimony trioxide	Ore processing; glass and ceramic production	Vol. 47 (1989c)	Inadequate	Sufficient
Cobalt and cobalt compounds	Miners; processing of copper and nickel ore; glass and ceramic production	Vol. 52 (1991a)	Inadequate	Sufficient
Lead and inorganic lead compounds	Lead smelters; plumbers; solderers; occupations in battery recycling smelters	Suppl. 7 (1987)	Inadequate	Sufficient
Methyl mercury compounds	Pesticide and fungicide production; paint industry	Vol. 58 (1993a)	Inadequate	Sufficient
Nickel: metallic and alloys	Nickel miners; metal fabrication, grinding, electroplating, and welding	Vol. 49 (1990a)	Inadequate	Sufficient
Wood and fossil fuels and their by-products				
Benzofuran	Production; intermediate in coumarone-indene resin polymerization; coke production; coal gasification and combustion	Vol. 63 (1995a)	Not available	Sufficient
Bitumens, extracts of steam-refined and air-refined	Production/refining; road construction; roofing and flooring	Suppl. 7 (1987)	Inadequate	Sufficient
Carbon black	Production; paint, ink, plastic and rubber industries	Vol. 65 (1996)	Inadequate	Sufficient
Diesel fuel, marine	Petroleum refineries; marine fuel; distribution	Vol. 45 (1989b)	Inadequate	Limited
Fuel oils, residual (heavy)	Petroleum refineries; distribution; marine fleets; most large diesel engines operated on land; industrial heating systems	Vol. 45 (1989b)	Inadequate	Sufficient
Gasoline	Petroleum refineries; transportation; mechanics and service station attendants	Vol. 45 (1989b)	Inadequate	Limited
Gasoline engine exhaust	Transportation and vehicle maintenance workers; drivers; toll attendants; traffic controllers	Vol. 46 (1989a)	Inadequate	Limited
Naphthalene	Production; insecticide, resin, and pharmaceutical production	Vol. 82 (2002b)	Inadequate	Sufficient
Polyaromatic hydrocarbons				
Benzo[*b*]fluoranthene	Work involving combustion of organic matter	Vol. 32 (1983b)	Not available	Sufficient
Benzo[*j*]fluoranthene	Work involving combustion of organic matter	Vol. 32 (1983b)	Not available	Sufficient
Benzo[*k*]fluoranthene	Work involving combustion of organic matter	Vol. 32 (1983b)	Not available	Sufficient
Dibenz[*a,h*]acridine	Production; used in dye synthesis; biochemical laboratory workers; work involving combustion of organic matter	Vol. 32 (1983b)	Not available	Sufficient
Dibenz[*a,j*]acridine	Production; dye synthesis; work involving combustion of organic matter	Vol. 32 (1983b)	Not available	Sufficient
Dibenzo[*a,e*]pyrene	Production; biochemical laboratory workers; work involving combustion of organic matter	Vol. 32 (1983b)	Not available	Sufficient
Dibenzo[*a,h*]pyrene	Production; biochemical laboratory workers; work involving combustion of organic matter	Vol. 32 (1983b)	Not available	Sufficient
Dibenzo[*a,i*]pyrene	Work involving combustion of organic matter	Vol. 32 (1983b)	Not available	Sufficient
Dibenzo[*a,l*]pyrene	Production; biochemical laboratory workers; work involving combustion of organic matter	Vol. 32 (1983b)	Not available	Sufficient
Monomers				
Acrylonitrile	Production; acrylic textile fiber and plastic production	Vol. 71 (1999a)	Inadequate	Sufficient
Chloroprene	Production; manufacture of polychloroprene (synthetic rubber)	Vol. 71 (1999a)	Inadequate	Sufficient
Ethyl acrylate	Production; plastic molding occupations using acrylate resins	Vol. 39 (1986a)	Not available	Sufficient
Isoprene	Production; synthetic rubber and plastics industries	Vol. 71 (1999a)	Not available	Sufficient
Styrene	Polyester resin manufacture; production of packaging materials and fiberglass-reinforced polyester	Vol. 82 (2002b)	Limited	Limited
Toluene diisocyanates	Production; production of polyurethane foams and wire coating; insulation workers; ship builders	Vol. 71 (1999a)	Inadequate	Sufficient
Urethane	Production; amino-resin production	Vol. 7 (1974a)	Not available	Sufficient
Vinyl acetate	Production; plastics, paint, and adhesive industries	Vol. 63 (1995a)	Not available	Limited
Intermediates in plastics and rubber manufacturing				
Acetaldehyde	Acetic acid production workers; dyestuff, plastic and synthetic rubber industries	Vol. 71 (1999a)	Inadequate	Sufficient
Acetamide	Production; plastics and chemical industries	Vol. 71 (1999a)	Not available	Sufficient
2,4-Diaminotoluene	Production; chemical intermediate in TDI production; dyes for textiles; leather; furs; wood; biologic stain; photo developer	Vol. 16 (1978)	Not available	Sufficient
1,2-Epoxybutane	Production; metal degreasing; plastics industry	Vol. 71 (1999a)	Not available	Limited
Ethylbenzene	Production; ink, paint, and plastic production	Vol. 77 (2000b)	Inadequate	Sufficient
Ethylene thiourea	Production; vulcanization in the rubber industry; manufacture of ethylenebisdithiocarbamate pesticides; electroplating baths; dyes; pharmaceuticals; synthetic resins	Vol. 79 (2001b)	Inadequate	Sufficient
Phenyl glycidyl ether	Production; epoxy resins; casting and molding	Vol. 71 (1999a)	Not available	Sufficient
Propylene oxide	Production; polyurethane foam and glycol production, fumigant	Vol. 60 (1994)	Inadequate	Sufficient
Chlorinated hydrocarbons				
Carbon tetrachloride	Production; industrial degreasing occupations; dry cleaners; refrigerant production	Vol. 71 (1999a)	Inadequate	Sufficient
Chlorinated paraffin of average carbon-chain length C12	Production; polyvinyl chloride processing industry	Vol. 48 (1990b)	Not available	Sufficient
Chloroform	Refrigerant production; dyes, solvents, and pesticides	Vol. 73 (1999b)	Inadequate	Sufficient
1,2-Dichloroethane	Vinyl chloride production workers	Vol. 71 (1999a)	Inadequate	Sufficient
Dichloromethane	Production; painters and furniture restorers; pharmaceutical and electronic production	Vol. 71 (1999a)	Inadequate	Sufficient
Hexachloroethane	Production; aluminum refinery; industrial firefighters	Vol. 73 (1999b)	Inadequate	Sufficient
Aromatic amine dyes				
Auramine (technical grade)	Production; textiles, plastic, and printing	Suppl. 7 (1987)	Inadequate	Sufficient
Benzyl violet 4B	Production; food; drugs; cosmetics; textiles	Vol. 16 (1978)	Not available	Sufficient
CI Basic Red 9	Production; textiles; printing; biologic stains (basic fuchsin dye in laboratories)	Vol. 57 (1993b)	Inadequate	Sufficient
2,4-Diaminoanisole	Dyestuff industry; barbers and cosmetologists; furriers	Vol. 79 (2001b)	Not available	Sufficient
3,3′-Dimethylbenzidine (*o*-tolidine)	Production; dye or intermediate in dye and pigment production; polyurethane elastomers; coating; plastics; clinical laboratories	Vol. 1 (1972)	Not available	Sufficient
2,6-Dimethylaniline (2,6-xylidine)	Production; dyestuffs and pharmaceutical manufacturing	Vol. 57 (1993b)	Not available	Sufficient
3,3′-Dichlorobenzidine	Production; dyestuff manufacturing	Vol. 29 (1982b)	Inadequate	Sufficient
4,4′-Diaminodiphenyl ether	Production; polyamide-type resin manufacturing	Vol. 29 (1982b)	Not available	Sufficient
Disperse Blue 1	Production; hair coloring; textiles and plastics	Vol. 48 (1990b)	Not available	Sufficient
HC Blue No. 1	Production; hair dye	Vol. 57 (1993b)	Not available	Sufficient
4,4′-Methylenedianiline	Production; production of diisocyanates, polyisocyanates, and epoxy resins	Vol. 39 (1986a)	Not available	Sufficient
Magenta containing CI Basic Red 9	Production; textiles and printing; biologic stains in laboratories; photography	Vol. 57 (1993b)	Not available	Sufficient
Azo dyes				
*ortho*-Aminoazotoluene	Production; textiles and leather	Vol. 8 (1975)	Not available	Sufficient
*para*-Aminoazobenzene	Production; textiles and leather	Suppl. 7 (1987)	Not available	Sufficient
CI Acid Red 114	Production; textiles and leather	Vol. 57 (1993b)	Not available	Sufficient
CI Direct Blue 15	Production; textiles and paper	Vol. 57 (1993b)	Not available	Sufficient
Citrus Red No. 2	Production; used for food coloring	Vol. 8 (1975)	Not available	Sufficient
*para*-Dimethylaminoazobenzene	Production; textiles; laboratories	Vol. 8 (1975)	Not available	Sufficient
Oil orange SS	Production; dyes/pigments for varnishes, oils, fats, and waxes	Vol. 8 (1975)	Not available	Sufficient
Ponceau 3R	Production; textiles	Vol. 8 (1975)	Not available	Sufficient
Ponceau MX	Production; textiles; leather; inks; paper; wood stains; food; biology laboratories	Vol. 8 (1975)	Not available	Sufficient
Trypan blue	Production; textiles and printing; biologic stains in life science laboratories; used by ophthalmologists	Vol. 8 (1975)	Not available	Sufficient
Intermediates for the manufacture of dyes				
*para*-Cresidine	Production; manufacture of dyes, pigments, and perfumes	Vol. 27 (1982a)	Not available	Sufficient
3,3′-Dimethoxybenzidine (*ortho*-dianisidine)	Production; manufacture of dyes and pigments; dye for leather, paper, plastics, rubber, textiles, and laboratories	Suppl. 7 (1987)	Inadequate	Sufficient
2-Methyl-1-nitro anthraquinone (of uncertain purity/impurity)	Production; synthesis of anthraquinone dyes	Vol. 27 (1982a)	Not available	Sufficient
4,4′-Methylene bis (2-methylaniline)	Production; manufacture of dyes and pigments	Suppl. 7 (1987)	Inadequate	Sufficient
2-Nitroanisole	Production; manufacture of the dye intermediates *ortho*-anisidine and *ortho*-dianisidine	Vol. 65 (1996)	Not available	Sufficient
4,4′-Thiodianiline	Production; manufacture of dyes	Vol. 27 (1982a)	Not available	Sufficient
Nitro compounds				
2,4-Dinitrotoluene	Production; manufacture of diisocyanates and munitions	Vol. 65 (1996)	Inadequate	Sufficient
2,6-Dinitrotoluene	Production; manufacture of diisocyanates and munitions	Vol. 65 (1996)	Inadequate	Sufficient
Nitrobenzene	Production; manufacture of dyestuffs, detergents, and cosmetics	Vol. 65 (1996)	Not available	Sufficient
2-Nitrofluorene	Underground miners using diesel-powered machinery	Vol. 46 (1989a)	Not available	Sufficient
2-Nitropropane	Production; ink, paint, explosives industries	Vol. 71 (1999a)	Not available	Sufficient
1-Nitropyrene	Production; manufacture of azidopyrene; particulate emissions	Vol. 46 (1989a)	Not available	Sufficient
4-Nitropyrene	Production; used only as a laboratory chemical; probably present before 1980 in carbon black used in photocopy machines	Vol. 46 (1989a)	Not available	Sufficient
Tetranitromethane	Production; diesel fuel additive; TNT manufacturing	Vol. 65 (1996)	Not available	Sufficient
Pesticides				
Aramite	Production; in miticides in greenhouses, nurseries, and orchards	Vol. 5 (1974b)	Not available	Sufficient
Chlordane	Production; termite control	Vol. 79 (2001b)	Inadequate	Sufficient
Chlordecone	Production; insecticide	Vol. 20 (1979a)	Not available	Sufficient
Chlorophenoxy herbicides	Production; defoliant	Suppl. 7 (1987)	Limited	Inadequate
Chlorothalonil	Production; fungicide, bactericide, and nematocide	Vol. 73 (1999b)	Not available	Sufficient
DDT (*p*,*p*′-DDT)	Production; nonsystemic insecticide	Vol. 53 (1991b)	Inadequate	Sufficient
1,2-Dibromo-3-chloropropane	Production; pesticide, nematocide, and soil fumigant	Vol. 71 (1999a)	Inadequate	Sufficient
*para*-Dichlorobenzene	Production; pesticide	Vol. 73 (1999b)	Inadequate	Sufficient
Dichlorvos	Production; insecticide and miticide	Vol. 53 (1991b)	Inadequate	Sufficient
Heptachlor	Production; termite control	Vol. 79 (2001b)	Inadequate	Sufficient
Hexachlorobenzene	Production; in chlorinated pesticides and fungicides; dye manufacture and synthesis of organic chemicals and rubber; plasticizer for polyvinyl chloride; wood preservative; by-product of the production of a number of chlorinated solvents	Vol. 79 (2001b)	Inadequate	Sufficient
Hexachlorocyclohexanes (most common form is Lindane)	Production; woodworkers; farm workers	Suppl. 7 (1987)	Inadequate	Sufficient
Mirex	Production; fire-retardant additive; insecticide; workers at hazardous waste sites	Vol. 20 (1979a)	Not available	Sufficient
Nitrofen	Production; herbicide	Vol. 30 (1983a)	Not available	Sufficient
Sodium *ortho*-phenylphenate	Production; fungicide; chemical intermediate	Vol. 73 (1999b)	Not available	Sufficient
Toxaphene (polychloronated camphenes)	Production; insecticide	Vol. 79 (2001b)	Inadequate	Sufficient
Others				
Butylated hydroxyanisole (BHA)	Production; food and pharmaceutical industries	Vol. 40 (1986b)	Not available	Sufficient
Catechol	Production; insecticide and pharmaceutical production; tanneries	Vol. 71 (1999a)	Not available	Sufficient
Diglycidyl resorcinol ether	Production; liquid spray epoxy resin in electrical, tooling, adhesive, and laminating applications; production of epoxy resins and rubber; aerospace industry	Vol. 71 (1999a)	Not available	Sufficient
1,4-Dioxane	Production; chlorinated solvents; textile processing; mixed with pesticides	Vol. 71 (1999a)	Inadequate	Sufficient
Hydrazine	Production; manufacture of agricultural chemicals and chemical blowing agents; water treatment; spandex fibers; rocket fuel; oxygen scavenger in water boilers and heating systems; scavenger for gases; plating metals on glass and plastics; solder fluxes; photographic developers; reactant in fuel cells in the military; reducing agent in electrodeless nickel plating; chain extender in urethane; textile dyes; explosives	Vol. 71 (1999a)	Inadequate	Sufficient
Nitrilotriacetic acid and its salts	Production; textiles; electroplaters; tanners	Vol. 73 (1999b)	Not available	Sufficient
Polychlorophenols and their sodium salts (mixed exposure)	Herbicide production; wood, textile and leather manufacturing	Vol. 71 (1999a)	Limited	Inadequate
Potassium bromate	Production; bakeries	Vol. 73 (1999b)	Not available	Sufficient
Thiourea	Production; photoprocessing; dyes; rubber industry	Vol. 79 (2001b)	Not available	Sufficient
Welding fumes	Metal fabricating industry	Vol. 49 (1990a)	Limited	Inadequate

TDI, toluene diisocyanate.

a Not necessarily an exhaustive list of occupations/industries in which this agent is found; not all workers in these occupations/industries are exposed. The term “production” is used to indicate that this substance is man-made and that workers may be exposed in the production process.

b Most recent IARC evaluation; for those referenced as Supplement 7 (IARC 1987), it is possible that the 1987 review was quite perfunctory and that the essential evidence was cumulated at an earlier date.

c As judged by the IARC working group; we added the notation “not available” to signify those substances for which there was no epidemiologic evidence at all.

**Table 6:** Occupations or industries that have been evaluated by IARC as definitely (group 1), probably (group 2A), or possibly (group 2B) entailing excess risk of cancer among workers.

Occupation or industry	Suspected substance	*IARC Monograph* volume (year)*^a^*	Group	Site(s)
Aluminum production	Pitch volatiles; aromatic amines	Suppl. 7 (1987)	1	Lung,*^b^* bladder*^b^*
Auramine manufacture	2-Naphthylamine; auramine; other chemicals; pigments	Suppl. 7 (1987)	1	Bladder*^b^*
Boot and shoe manufacture and repair	Leather dust; benzene and other solvents	Suppl. 7 (1987)	1	Leukemia,*^b^* nose,*^b^* paranasal sinuses,*^b^* bladder*^c^*
Carpentry and joinery	Wood dust	Suppl. 7 (1987)	2B	
Coal gasification	Coal tar; coal-tar fumes; PAHs	Vol. 34 (1984)	1	Skin (including scrotum),*^b^* bladder,*^b^* lung*^b^*
Coke production	Coal-tar fumes	Suppl. 7 (1987)	1	Skin (scrotum),*^b^* lung,*^b^* bladder,*^c^* kidney*^c^*
Dry cleaning	Solvents and chemicals used in “spotting”	Vol. 63 (1995a)	2B	
Furniture and cabinet making	Wood dust	Suppl. 7 (1987)	1	Nose and sinonasal cavities*^b^*
Hairdressers and barbers	Dyes (aromatic amines, amino-phenols with hydrogen peroxide); solvents; propellants; aerosols	Vol. 57 (1993b)	2A	Bladder,*^c^* lung,*^c^* non-Hodgkin lymphoma,*^c^* ovary*^c^*
Hematite mining, underground, with radon exposure	Radon daughters; silica	Suppl. 7 (1987)	1	Lung*^b^*
Iron and steel founding	PAHs; silica; metal fumes; formaldehyde	Suppl. 7 (1987)	1	Lung*^b^*
Isopropanol manufacture, strong-acid process	Diisopropyl sulfate; isopropyl oils; sulfuric acid	Suppl. 7 (1987)	1	Paranasal sinuses,*^b^* larynx,*^b^* lung*^c^*
Magenta manufacture	Magenta; *ortho*-toluidine; 4,4′-methylene bis(2-methylaniline); *ortho*-nitrotoluene	Vol. 57 (1993b)	1	Bladder*^b^*
Painters		Vol. 47 (1989c)	1	Lung,*^b^* bladder,*^c^* stomach*^c^*
Petroleum refining	PAHs	Vol. 45 (1989b)	2A	Bladder,*^c^* brain,*^c^* leukemia*^c^*
Printing processes	Solvents; inks	Vol. 65 (1996)	2B	
Production of art glass, glass containers, and pressed ware	Lead; arsenic; antimony oxides; silica; asbestos; other metal oxides; PAHs	Vol. 58 (1993a)	2A	Lung*^c^*
Rubber industry	Aromatic amines; solvents	Suppl. 7 (1987)	1	Bladder,*^b^* stomach,*^c^* larynx,*^c^* leukemia,*^c^* lung*^c^*
Textile manufacturing industry	Textile dust in manufacturing process; dyes and solvents in dyeing and printing operations	Vol. 48 (1990b)	2B	

a Most recent IARC evaluation; for those referenced as Supplement 7 (IARC 1987), it is possible that the 1987 review was quite perfunctory and that the essential evidence was cumulated at an earlier date.

b We judged that the evidence for an association with this site was strong.

c We judged that the evidence was suggestive.

**Table 7:** Definite or probable occupational carcinogens and carcinogenic circumstances, by site.

Site	Strength of evidence*^a^*	High-risk substance or circumstance
Pharynx and nasopharynx	Suggestive	Mustard gas; formaldehyde
Nasal cavities and paranasal sinuses	Strong	Boot and shoe manufacture and repair; furniture and cabinet making; isopropanol manufacture, strong acid process; selected nickel compounds, including combinations of nickel oxides and sulfides in the nickel-refining industry; wood dust
	Suggestive	Chromium compounds, hexavalent; formaldehyde; mineral oils, untreated and mildly treated
Esophagus	Suggestive	Soots; tetrachloroethylene
Stomach	Suggestive	Painters; rubber industry
Gastrointestinal tract	Suggestive	Asbestos
Liver and biliary tract	Strong	Aflatoxin; ionizing radiation
	Suggestive	Polychlorinated biphenyls; trichloroethylene
Liver (angiosarcoma)	Strong	Vinyl chloride
	Suggestive	Arsenic and arsenic compounds
Liver (hepatocellular)	Suggestive	Vinyl chloride
Pancreas	Suggestive	Acrylamide
Larynx	Strong	Isopropanol manufacture, strong acid process; inorganic acid mists containing sulfuric acid; mustard gas
	Suggestive	Asbestos; rubber industry
Lung	Strong	Aluminum production; arsenic and arsenic compounds; asbestos; beryllium; cadmium and cadmium compounds; chromium compounds, hexavalent; coal gasification; coke production; hematite mining, underground, with radon exposure; involuntary (passive) smoking; ionizing radiation; iron and steel founding; selected nickel compounds, including combinations of nickel oxides and sulfides in the nickel refining industry; painters; silica, crystalline; soots; talc containing asbestiform fibers
	Suggestive	Benz[*a*]anthracene; benzo[*a*]pyrene; α-chlorinated toluenes; coal tars and pitches; dibenz[*a,h*]anthracene; diesel engine exhaust; epichlorohydrin; hairdressers and barbers; inorganic acid mists containing sulfuric acid; isopropanol manufacture (strong acid process); mineral oils (untreated and mildly treated); nonarsenical insecticides; mustard gas; production of art glass, glass containers, and pressed ware; rubber industry; TCDD
Lung (oat cell)	Strong	Bis(chloromethyl) ether and chloromethyl methyl ether (technical grade)
Bone	Strong	Ionizing radiation
Melanoma	Strong	Solar radiation
	Suggestive	Ultraviolet radiation (A, B and C) from artificial sources
Skin	Strong	Arsenic and arsenic compounds; Coal tars and pitches; coal gasification; coke production; dibenz[*a,h*]anthracene; mineral oils, untreated and mildly treated; shale oils or shale-derived lubricants; solar radiation; soots
	Suggestive	Benz[*a*]anthracene; benzo[*a*]pyrene; creosotes
Mesothelioma	Strong	Asbestos; erionite; talc containing asbestiform fibers
CNS	Suggestive	Epichlorohydrin
Sarcoma	Suggestive	TCDD
Cervix	Suggestive	Tetrachloroethylene
Ovary	Suggestive	Hairdressers and barbers
Kidney	Suggestive	Coke production
Kidney (renal cell)	Suggestive	Trichlorethylene
Bladder	Strong	Aluminum production; 4-aminobiphenyl; auramine manufacture; benzidine; coal gasification; magenta manufacture; 2-naphthylamine; rubber industry
	Suggestive	Benz[*a*]anthracene; benzidine-based dyes; benzo[*a*]pyrene; boot and shoe manufacture and repair; 4-chloro-*ortho*-toluidine; coal tars and pitches; coke production; dibenz[*a,h*]anthracene; diesel engine exhaust; hairdressers and barbers; 4,4′-methylene bis(2-chloroaniline); mineral oils, untreated and mildly treated; *ortho*-toluidine; painters; petroleum refining
Brain	Suggestive	Nonarsenical insecticides; petroleum refining
Thyroid	Strong	Ionizing radiation
Non-Hodgkin lymphoma	Suggestive	Hairdressers and barbers; nonarsenical insecticides; TCDD; tetrachloroethylene; trichloroethylene
Lympho-hematopoietic system	Suggestive	1,3-Butadiene
Multiple myeloma	Suggestive	Nonarsenical insecticides
Leukemia	Strong	Benzene; boot and shoe manufacture and repair; ethylene oxide; ionizing radiation
	Suggestive	Formaldehyde; nonarsenical insecticides; petroleum refining; rubber industry
Other sites	Suggestive	Ionizing radiation*^b^*
All sites combined	Strong	TCDD*^c^*

CNS, central nervous system; TCDD, 2,3,7,8-tetrachlorodibenzo-*para*-dioxin.

a Our judgment of strength of evidence regarding each site.

b There is suggestive evidence of an effect of ionizing radiation on several sites in addition to those shown here.

c The evidence for an association with TCDD only becomes strong when data are combined for all cancer sites.

**Table 8:** Evolution in knowledge regarding current (2003) IARC occupational carcinogens.

	Earlier evaluation		
Current rating	Past rating	IARC 1987	WHO 1964
1 (*n* = 28)	1	19	13
	2A	4	4
	2B	1	
	3	0	NA
	Unrated	4	11
	Total	28	28
2A (*n* = 27)	1	0	0
	2A	16	0
	2B	6	
	3	2	NA
	Unrated	3	27
	Total	27	27
2B (*n* = 113)	1	0	1
	2A	2	5
	2B	63	
	3	9	NA
	Unrated	39	107
	Total	113	113

NA, not applicable.
