# Errata

**Published:** 2005-02

Siemiatycki et al. have detected some errors in their review of occupational carcinogens published in the November 2004 issue of *EHP* [ Environ Health Perspect 112:1447–1459 (2004)]. Specifically, they inadvertently included in their list of Group 2B (possible) carcinogens some substances that had been downgraded to Group 3 (not classifiable) in a subsequent *IARC* (International Agency for Research on Cancer) *Monograph*. Their corrections are as follows:

- On page 1449, 3rd column, “113 possible human occupational carcinogens (IARC Group 2B; Table 5)” should be replaced by “110 possible human occupational carcinogens (IARC Group 2B; Table 5).”
- On page 1454, Table 5 (under “Respirable dusts and fibers”), glass wool, rock wool, and slag wool fireproofing should not have appeared in the listing of Group 2B human carcinogens because they were downgraded to Group 3 in the latest monograph to address these substances (IARC 2002a); special purpose glass fibers such as E-glass and “475” glass fibers are not used in the “Reinforced plastic industry” but rather in “High-efficiency air filtration media and battery separator media” (IARC 2002a). A corrected version of this section of Table 5 is presented below.
- On page 1459, Table 8, as a result of the previous corrections, the last section of Table 8 (Current rating 2B) should be modified as follows: the total number of substances with this rating should read 110 instead of 113; the number of substances unrated by IARC in 1987 should read 36 instead of 39; and the number of substances unrated by the World Health Organization (WHO) in 1964 should read 104 instead of 107. A corrected version of Table 8 is presented below.

Siemiatycki et al.’s review of the *IARC Monographs* was intended to cover volumes 1–83. With these corrections, the tables and text are complete.

## Figures and Tables

**Table 5:** Substances and mixtures that have been evaluated by IARC as possible (Group 2B) human carcinogens and that are occupational exposures [corrected section only].

Substance or mixture	Occupation or industry in which the substance is found^a^	IARC Monograph volume (year)^b^	Human evidence^c^	Animal evidence^c^
Respirable dusts and fibers				
Palygorskite (long fibers > 5 μm)	Miners and millers; production of waste absorbents, fertilizers, and pesticides	Vol. 68 (1997b)	Inadequate	Sufficient
Refractory ceramic fibers	Production; furnace insulators; ship builders; heat-resistant fabric manufacture	Vol. 81 (2002a)	Inadequate	Sufficient
Special-purpose glass fibers such as E-glass and “475” glass fibers	High-efficiency air filtration media; battery separator media	Vol. 81 (2002a)	Not available	Sufficient

**Table 8:** Evolution in knowledge regarding current (2003) IARC occupational carcinogens (corrected version).

	Earlier evaluation			
Current rating	Past rating	IARC 1987		WHO 1964
1 (*n* = 28)	1	19		13
	2A	4	]—	4
	2B	1	
	3	0		NA
	Unrated	4		11
	Total	28		28
2A (*n* = 27)	1	0		0
	2A	16	]—	0
	2B	6	
	3	2		NA
	Unrated	3		27
	Total	27		27
2B (*n* = 110)	1	0		1
	2A	2	]—	5
	2B	63	
	3	9		NA
	Unrated	36		104
	Total	110		110

NA, not applicable.
